# Navigating the Caregiving Pathway: Understanding the Contextual Influences on Sense of Coherence Among Family Caregivers

**DOI:** 10.7759/cureus.57815

**Published:** 2024-04-08

**Authors:** Arsha Kochuvilayil, Ravi P Varma

**Affiliations:** 1 Epidemiology and Public Health, Achutha Menon Centre for Health Science Studies, Sree Chitra Tirunal Institute for Medical Sciences & Technology, Thiruvananthapuram, IND

**Keywords:** meaningfulness, manageability, comprehensibility, family caregivers, caring roles, sense of coherence

## Abstract

Introduction: Family caregivers of patients with chronic conditions face challenges such as emotional and physical stress, which can lead to caregiver burden. A good sense of coherence (SOC) is crucial in promoting resilience, positive health outcomes, and coping. Caregivers with a high SOC are optimistic about their caregiving roles and finding meaning and purpose in their responsibilities. In this background, we looked into the contextual influences that facilitate or impede the sense of coherence of the family caregivers of patients with chronic conditions requiring home-based long-term care.

Methods: We conducted telephonic interviews with 10 self-identified primary family caregivers of patients with chronic conditions. We utilized semi-structured interview guidelines, transcribed the interviews verbatim, and performed thematic analysis. Potential factors influencing caregivers' SOC were identified through inductive coding, allowing themes to emerge from the data. However, we report themes along the three components of SOC.

Results: Good knowledge about the disease conditions, open communication with care recipients and providers, and past caregiving experiences all contribute to improving comprehensibility. Insufficient knowledge about the condition could be distressing. Effective management requires adapting care strategies through learning, planning, and utilizing available resources, and support networks, too, play a crucial role. However, insufficient caregiver support and neglecting one's health can result in distress and disruptions in care management. Maintaining positive perspectives and ascribed values to interpersonal connections can enhance meaningfulness among caregivers. These interpretations may not apply to caregivers with affective disorders.

Conclusion: Various aspects influence the comprehensibility, manageability, and meaningfulness pertaining to the situation of family caregivers, and these in turn impact their well-being and ability to provide quality care. Understanding these factors can help create support systems with targeted interventions and strategies to reduce caregiver burden and improve quality of life.

## Introduction

The aging population and rising life expectancy necessities the need for long-term care for the elderly and people with chronic conditions. Informal caregivers, often women family members, meet these care needs [1]. Such caregivers face numerous challenges, including emotional and physical stress and strain that can lead to or exacerbate caregiver burden [2-4]. Care tasks are heavily gendered, and most family caregivers are women. Traditional gendered roles and norms mean that women have much less power in decision-making than men within domestic spaces. Caregivers may thus have to negotiate with complex challenges and may end up feeling helpless and hopeless. Caregivers' incapability of supporting the care recipient may be associated with feelings of failure, anxiety, guilt, and unmet needs. These feelings can be a significant part of the caregiving experience and can be detrimental to both the caregiver and the care recipient - a "burning at both ends" situation marked with feelings of helplessness and hopelessness [5].

Research into negotiating with these roles and their challenges increasingly focuses on the concept of sense of coherence (SOC). A person's SOC is significant for promoting resilience, positive health outcomes, and adaptive coping across diverse populations. A high level of SOC protects against anxiety, depression, post-traumatic stress disorder, and burnout or exhaustion [6-7]. Those who have a greater sense of SOC have a higher quality of life and can also significantly predict a caregiver's emotional well-being [8]

According to Eriksson, SOC, as developed by Antonovsky, is a salutogenic concept that can explain why certain individuals remain healthy while others become sick during hardship [9]. SOC centers around the concept of health and well-being and demonstrates how an individual's perspective on life and capability can influence how they deal with challenging circumstances. It involves seeing life as ordered, manageable, and meaningful. It describes an individual method of using their inner trust, a compassionate concern for themself and their abilities, which enables them to find, take advantage of, utilize, and repurpose the resources available to them. SOC thus has three components: comprehensibility, manageability, and meaningfulness [10]. These components constitute a theoretical model for understanding the relationship between stress, coping, and health. SOC is a salutogenic concept that refers to the common attribute underlying the utilization of resources and seeing the world positively to attain adaptive coping with complex stressors [11]. Comprehensibility can be seen as a cognitive attribute that enables the individual to comprehend their internal and external circumstances rationally. Manageability is a behavioral attribute that indicates managing and utilizing resources to deal with a situation. Meaningfulness can be seen as a motivating attribute that gives particular emotional meaning to our lives, which helps us to navigate through everyday problems or challenges. Caregivers with a high SOC tend to be optimistic about their caregiving duties, finding meaning and purpose in their responsibilities and making it easier to deal with the challenges that come with it. They better understand the care recipient's needs, enabling them to provide better care and assistance. Thus, having a high SOC can serve as a tool for efficient coping among caregivers [12].

Several studies reiterate the favorable impact of high SOC levels on family caregivers on their health-related aspects, psychological and coping aspects, and negative influence on the burden experienced by them [13-15]. A caregiver's level of SOC may be shaped by multiple aspects steeped within the contexts where the caring role emerges and is sustained. Biddle's role theory, a symbolic interactionist approach that permits some active place for individuals, considers roles as evolving through the interplay of individual and social processes that we refer to as the context here. For instance, values may be considered as individual-level aspects but inevitably reflect social concept systems like body-related, economic, or religious norms and ideologies, even if individual-level interpretations may conveniently alter these. This theory works well in small social systems like within families [16]. There is limited evidence on the contextual influences that shape the SOC among such family caregivers. Therefore, we conducted an exploratory study among family caregivers of patients with chronic conditions on the contextual influences that potentially facilitate or impede their sense of coherence. Understanding these can aid in creating support systems that enhance health outcomes and well-being for family caregivers, enabling targeted interventions and strategies to reduce burden and improve quality of life.

An initial analysis of this work was presented at the sixth Amrita International Public Health Conference, which was held on December 1-2, 2023.

## Materials and methods

Research design

This study was carried out as part of a formative assessment of care recipient suffering and caregiving experiences of caregivers of patients with chronic conditions that require long-term care support. We used in-depth interviews to delve into the caregiving experience of family caregivers. Recognizing the subjective nature of caregiving and its intricate ties to social, cultural, and political contexts, we opted for in-depth interviews to investigate this phenomenon.

Researcher characteristics and reflexivity

Both researchers hold postgraduate degrees in public health or community medicine and have extensively explored constructivist and post-positivist ideas in their research on palliative care. This familiarity with these approaches and various study findings formed the foundation for developing the in-depth interview guidelines and analyzing the interview data. AVR, who conducted the interviews and did the initial analysis, lacks direct experience in providing palliative care but has close engagement with caregivers for about five years and identifies as a woman from within the same cultural setting as the caregivers interviewed. Both authors were closely involved in caregiving within their respective families, which may have influenced their analysis due to personal experiences with the issue. However, we acknowledge that this background may not fully capture the intricacies of caregivers' experiences.

Context

The study was conducted in purposively selected panchayats of Kollam district, Kerala. The state-run palliative care home program has been implemented in all panchayats. Under this, palliative care nurses offer monthly home visits to chronically ill patients confined to their homes, ensuring practical and immediate care and support for accomplishing activities of daily living.

Sampling strategy

At first, palliative care patients were selected purposively from the chosen panchayats of the Kollam district through palliative care nurses and Accredited Social Health Activists (ASHA) of the respective panchayats. Patients with cancer, dementia, heart failure, chronic kidney disease, and stroke were selected purposively to get a range of caregiver experiences. These conditions constitute the top five chronic morbidities according to the Global Burden of Disease study estimates for India [17]. The caregiving requirements for these are likely to be long-term and diverse. For each patient chosen, one self-identified adult (more than 18 years) primary caregiver providing care for at least 12 months was included. Based on descriptions of potential participants elicited from the palliative care nurses or ASHA workers by AVR, cares who were physically or emotionally unable to participate or whose care recipient was critically ill at the time of the interview were excluded. The sample limit of 10 depended on reaching the thematic saturation during the analysis of in-depth interviews.

Data collection methods

In-depth interviews were used to capture the care experiences of the family caregivers. A semi-structured question set with probes was used to conduct the in-depth interviews. The interviewer used prompts or questions to get participants to elaborate on their response - for instance, by eliciting the back story of a certain situation or requesting participants to elaborate further on some statements that the interviewer felt were relevant to the study. The interview was done telephonically at a mutually convenient time for the interviewer and the participant. AVR initially contacted prospective participants, explained the study's purpose, and took verbal consent, which was subsequently documented electronically when interviews were recorded. The interviews were conducted in the participants' native language (Malayalam).

Data analysis

All interviews were translated into English by the researcher before analysis. Open coding analysis was done manually. Emerged codes are mapped into categories to develop themes. The themes were then subsequently grouped to describe various aspects that facilitate and impede the three dimensions of SOC in family caregivers. AVR did the initial coding, followed by code refining and discussion with RPV. Discussion and recording were repeated until the two researchers reached a consensus. The above method helped clarify, illustrate, and validate the emerging patterns. To ensure an accurate understanding of the findings, direct excerpts from the interviews were used to explain the themes and categories that emerged. We tried to establish the dependability and confirmability of our findings through reflexive journaling, audit trails, periodic meetings between the investigators, and presentation of the findings to other researchers.

Ethical clearance

Ethics clearance was obtained from the Institutional Ethics Committee of Sree Chitra Tirunal Institute for Medical Sciences and Technology (SCTIMST) (Ref No. SCT/IEC/2048/May/2023).

## Results

Participant profiles

The average age of caregivers (CG) was 47.5 years old, ranging from 38 to 59 years old. All interviewed caregivers were women, spouses, or daughters/in-laws of the care recipients (CR). The average age of care recipients was 66.1 years old, ranging from 53 to 82 years old. The average duration of caregiving was 61.8 months. Interviews conducted for the study had an average duration of 30 minutes, though varied between 20 to 50 minutes (Table 1). We then report the themes that emerged from the analysis using illustrative participant quotes. We have partially modified the quotes for clarity and ease of understanding, and these are indicated using ellipses or angle brackets. Each quote is followed by the diagnosis of the care recipient, the age of the caregiver in years, and the relationship of the caregiver to the care recipient in brackets.

**Table 1:** Participant Profile M: Male; F: Female

Patient age (years)	Patient sex	Caregiver age (years)	Caregiver sex	Duration of care (months)	Condition of the patient	Relation to the patient	Employment status
60	F	38	F	72	Cancer	daughter	Beautician
58	M	51	F	60	Chronic Kidney Disease	spouse	Homemaker
64	M	59	F	24	Dementia	spouse	Cook
80	F	39	F	12	Dementia	daughter in law	Homemaker
80	F	41	F	12	Dementia	daughter in law	Homemaker
59	M	54	F	18	Heart Failure	spouse	Homemaker
53	M	47	F	216	Heart Failure	spouse	Homemaker
62	M	58	F	120	Stroke	spouse	Homemaker
63	M	39	F	72	Stroke	daughter	Homemaker
82	F	49	F	12	Stroke	daughter	Homemaker

Most caregivers expressed complex challenges related to the care role where they had to balance self-care, other responsibilities, and the care role-related tasks.

"…I will go (for my check-up). I will ask my kids to look after her (care recipient) ... I will go at that exact time of the appointment and come back soon after. I need to make time for myself... At night, she keeps calling and won't sleep. It's been like that for quite some time now. I need to get up early, also. It is difficult. Children <need to go> to college, so I have to make food for them. … Some issues are there. We are human, and we adapt to everything, so I'm doing all this. Our mind is everything. We will have that confidence. I became more adaptable to this over time." (Stroke CG, 49, daughter)

Caregivers respond to these challenges try to overcome them and the SOC level could be considered high when they succeed.

"…Earlier, he felt so bad that when I cleaned him after using the commode or bathed or dressed him. His face will be sad and all. I could feel that, but he never said anything. Then I will say something funny and make him laugh; like that, I manage him well. Now he is okay. I make him laugh while bathing to overcome his embarrassment." (Stroke CG, 39, daughter)

One caregiver could successfully balance her career as a beautician with control over her own routines and care-related tasks.

"… It's (the beauty parlor where the CG worked) near my home... I have kept a home nurse to take care of her when I'm away. She (home nurse) needs to do <very little>, only to give her the lunch through the <nasogastric> tube. If I need to go anywhere, she will come and sit with my mother... I will come back soon. I will cook and do everything… I won't stay anywhere overnight. I came back in 2-3 hours." (Cancer CG, 38, daughter)

Some others were struggling to cope with the situation.

"…I feel bad when she won't listen to me...I will ask her...' why you are doing this to me. Why you are like this to me?' I especially feel bad when someone comes home... If someone is visiting, I will get angry at her (care recipient) and make her change her clothes and <diapers>. She won't listen. Then I feel bad that I'm doing everything and she is behaving like that. I feel bad about that." (Dementia, CG, 41, daughter-in-law)

We have listed the emerging themes and elaborated them in the following section (Table 2). We have grouped the emerging themes and reported them under the logically consistent domain of SOC - comprehensibility, manageability, or meaningfulness - respectively. The themes represent enabling and hindering factors of various components of SOC and suggestive implications on the caregiver's capacity to cope with the caring role and avoid burden and distress. We have used the term care recipient instead of patient unless the statement involved primarily biomedical aspects of care.

**Table 2:** Themes indicating contextual aspects that may facilitate or impede a sense of coherence

Comprehensibility 1. Accessibility and knowledge about patient condition and previous experience 2. Access to formal and informal financial assistance and support 3. Open communication and guidance from health care providers and caregivers going through similar conditions 4. Logical beliefs about the care recipient's condition 5. Continuous learning and education about care recipient’s conditions and care needs and having a positive attitude towards the role 6. Lack of guidance, support, Knowledge about patient condition, and sole caregiving	Manageability 1. Planning and implementing diverse care approaches brings balance to care responsibilities and other responsibilities 2. Financial planning, management, and utilization of available resources 3. Previous experiences, support, and guidance from peers. 4. Ignoring personal health and self-care management 5. Lack of support and opportunity cost 6. Inadequate knowledge about the patient's specific care needs
	Meaningfulness 1. Caregiver's motivations 2. Lack of meaningful relationships and affective disorders

Aspects that facilitate and impede comprehensibility among family caregivers

Accessibility and Knowledge About Patient Condition and Previous Experience

Access to information about the patient's condition through healthcare professionals and personal networks enabled a more comprehensive approach to caregiving. Furthermore, obtaining and using information from friends provided valuable insights, such as alternative treatment facilities that offer better care at a reduced cost. This collective knowledge and experience facilitate the development of a comprehensive understanding of the situation, thereby helping caregivers make informed decisions about the provision of care. Previous experience in caregiving and firsthand knowledge of similar conditions within the family also contributed to a significantly enhanced understanding of the care required and its underlying circumstances. For one participant, having worked as an attendant in a hospital and the resultant familiarity with different care needs provided practical skills and insight into recipient care needs. "…my father died of kidney failure. Then my grandmother died of cancer. So, I didn't feel anything (about the cancer diagnosis of my mother). I know about the condition. Then my mother was also brave. She said that even small kids have cancer and I'm old so it is okay. She also took things bravely. My father also had (heart) attacks...3 times so I wasn't shocked" (Cancer Caregiver, 38, Daughter)

Access to Formal and Informal Financial Assistance and Support

Medical emergencies and chronic treatment require access to financial support and schemes. This can come from various formal and informal sources, such as bank loans, government schemes, family assistance, and help from friends and neighbors. Government schemes and insurance may provide financial aid or subsidized treatment options, easing the burden on patients and their families. Family and friends' support plays a significant role, often stepping in financially; most often, elderly caregivers heavily rely on their children for financial support. "There is a [socioeconomic category] in [Hospital name]. So we need only less money for treatment. But we need to submit our ration card and other documents. Then they [Hospital staff] considered us as [Socioeconomic category]. Because of that, we only need to spend about one-third of the amount" (Heart failure Caregiver, 54, Spouse)

Open Communication and Guidance From Healthcare Providers and Caregivers Going Through Similar Conditions

Open communication and guidance from healthcare providers (HCPs) and others are crucial in ensuring the appropriate care, such as wound care after surgery. Through this effective communication, caregivers can comprehensively understand their care recipient's condition, medications, and care needs. Additionally, interaction with other caregivers facing similar circumstances may lead to a gain of practical advice. "…they were helpful; they talked about my mother's condition… explained everything. My mother received good treatment." (Cancer Caregiver, 38, Daughter) *Logical Beliefs About the Care Recipient’s Condition*

Caregivers have diverse beliefs about the care recipient's condition, considering biological, environmental, and lifestyle factors. A comprehension of the cause of the disease or condition that makes sense and is perceived as logical by the caregiver provides a foundation for a good caregiving environment and the caregiver's ability to adjust to challenging circumstances. "The doctor said this was because of the intake of excessive medicines. Then the doctor said at that time not to take even one paracetamol for any cold or fever. Then we didn't take any medication after that." (CKD Caregiver, 51, Spouse) "He was a smoker. It is because of that. I used to tell him not to smoke. But he never listens. Only because of that, this happened." (Heart failure Caregiver, 47 Spouse) Continuous learning and education about patient conditions and care needs and having a positive attitude towards the role Continuous learning and education are fundamental components of providing quality patient care. By actively monitoring and understanding patient symptoms and medication, caregivers can easily understand patient disease progression and evolving care needs. Maintaining a positive attitude fosters a clearer understanding of circumstances and enables one to approach challenges with a constructive mindset. *Lack of Guidance, Support, and Knowledge About Care Recipient Condition and Sole Caregiving*

Caregivers may struggle to understand their situation without an adequate awareness and understanding of their care recipient's condition. Moreover, the absence of caregiver support from healthcare professionals further compounds these difficulties, as individuals may struggle to access the necessary resources and assistance to cope with their environment. Also, being the sole caregiver within such a dynamic can exacerbate feelings of isolation and be overwhelming, leading to increased emotional strain. "I'm sad about his condition. I'm struggling with him. I'm taking care of everything. I cannot do anything or cannot go out. Everything seems bad in my life. I always feel I'm alone." (Dementia Caregiver, 59, Spouse)

Aspects that facilitate and impede the manageability among family caregivers

Planning and Implementing Diverse Care Approaches Brings Balance to Care and Other Responsibilities

Meticulous planning and proactive measures can greatly contribute to the manageability of both patient care and other responsibilities. Caregivers may schedule hospital appointments well in advance to avoid long hospital wait periods. They also implement care approaches and behavior modification strategies like planning household activities to coincide with the care recipient's sleeping schedule or physical adjustments to the house to accommodate their specific needs, ensuring their comfort and safety. This may include modifications such as keeping doors closed to prevent the care recipient from wandering off, building attached toilets, and making changes to their clothing to ensure they are appropriately dressed. Establishing routines in care tasks along with close monitoring of recipient's condition helps to ensure effective care management.

Financial Planning, Management, and Utilization of Available Resources

Caregivers have adopted various strategies to manage household expenses while ensuring proper treatment for the patient. They have prioritized the financial resources for the patient's treatment by cutting down on non-essential household costs. Additionally, choosing treatment, medicines, and assistive aids provided by public services when available had helped minimize treatment expenses. The utilization of government schemes and insurance are valuable resources that help caregivers with budgeting, especially during extreme financial difficulties. Furthermore, having a job enabled such caregivers to contribute financially to the patient's care, supplementing available resources. "We decided to adjust. "Mundu murukki udutha appo jeeviche" (similar to "tightening our belts to survive"). (Stroke Caregiver, 58, Spouse)

Previous Experiences, Support, and Guidance From Peers

The caregiver's previous experiences and exposure to similar roles resulted in delivering better care and managing various responsibilities effectively. Patient-centered care plans, the caregiver's experience, and guidance from other caregivers in similar situations could improve manageability for family caregivers. Guidance from individuals who have shared similar caregiving experiences has been instrumental in shaping care approaches. Other family members offering assistance with caregiving responsibilities helped caregivers to better balance their lives with caregiving. Having a network of family and friends with whom the caregivers could share their emotional distress or challenges encountered in their caregiving role has been crucial for maintaining their well-being and resilience. Utilizing paid care support had been essential in providing some flexibility and enabling such caregivers to effectively balance their caregiving responsibilities with other commitments, such as their jobs. "I used to work in a hospital as an attendee. So, I know something (about the care needs)" (Heart failure Caregiver, 47, Spouse)

Ignoring Personal Health and Self-Care Management

Caregivers often neglect to monitor or seek care for their own health conditions due to their caregiving responsibilities. By failing to prioritize their well-being, caregivers risk their own health. Additionally, caregivers might conceal their illnesses from the care recipient and other family members, often to avoid incurring treatment costs in addition to the ongoing care recipient related expenses. "When he became sick... when his condition became bad... I gave more importance to him than me. I didn't buy (my) medicines. I put mine pending. I did nothing… no testing, no medicines, nothing. If he feels like I'm also sick, then he will be tense." (Heart failure Caregiver, 54, Spouse)

Lack of Support and Opportunity Cost

Lack of support impacted care task management and incurred an opportunity cost for caregivers. Lack of support often forced the caregiver to stop patient treatment, like physiotherapy, while they were forced to quit their job or lost their jobs and were no longer able to enjoy a social life. The absence of financial security and support further pushed them into debt. Sometimes, caregivers felt that the other family members were not dependable, choosing not to entrust them with care tasks.

Inadequate Knowledge About the Patient’s Specific Care Needs

Caregivers who lacked the knowledge and access to specific resources necessary for effectively managing the specific behaviors exhibited by their patients, like in dementia, made the management of care more difficult. This lack of awareness often led to difficult situations like controlling behaviors and inadequate emotional support.

Aspects that facilitate and impede meaningfulness among family caregivers

Caregiver's Motivations

Caregiver motivations include reciprocity, beliefs in meaningful relationships, and other benefits that foster caregiving. These motivations encompass various aspects that contribute to the meaning of caregiving. Key aspects here include a sense of appreciation from others, satisfaction, and happiness. Moreover, an increased understanding, compassion, and empathy within the care relationship create a profound sense of purpose. Among the perceived benefits that caregivers see as positive outcomes include the hope that their children will reciprocate the care in the future, personal growth and development, a positive outlook on life, and various religious and cultural beliefs. When all these aspects were aligned, it enabled caregivers to find profound meaningfulness in caregiving. "The doctor appreciated me "midukki" (good girl). He (patient) had become well physically "nannayi, kuttapanayi" (gained weight and became handsome)" (Stroke Caregiver, 58, Spouse) "Tomorrow I also will age. My children are seeing this and growing up." (Dementia Caregiver, 41, Daughter in law) "Now it is my turn to take care of her. I have to do that for my mother. we need to take care of her. It's our responsibility. We cannot expect anything in return for that…. This is our karma." (Stroke Caregiver, 49, Daughter) Lack of meaningful relationships and affective disorders Caregivers may lose meaningfulness due to feelings related to a lack of fulfillment, worsening of affective disorders, or loss of previous belief. When the caregiver-care recipient relationship lacks meaning, there is a lack of significance in the caregiving role and lives. "He stares at me like a stone. That's all. He won't comfort me or anything. He will do nothing…he just stares…nothing." (Stroke Caregiver, 58, Spouse)

## Discussion

Through a set of in-depth interviews, we aimed to learn about contextual influences that facilitate and impede the sense of coherence among family caregivers of palliative care patients in Kollam, Kerala. Being biomedically trained public health professionals with some research engagement with caregiving, we tried to list interview questions and generate conversations based on our readings and experience. Although it was difficult to claim an insider position alongside women caregivers who were primarily homemakers in Kollam, Kerala, we actively tried to situate our codes and themes about context aspects shaping SOC within participant narratives, which we then connected with the components of SOC. Participants might have chosen to give socially desirable responses to our inquiries about their roles and tasks. However, our efforts were to interpret aspects of the context where responses to difficult tasks associated with such roles emerged, and our findings would still be credible despite this. Our interpretation of context appears individualistic partly because of the small system where caregiving roles are evolving - the family level care - and partly because of the underlying symbolic interactionist perspective of Biddle's role theory that strongly retains individual-level aspects and places interplay with the context as the basis for evolution of roles. Caregivers start by appreciating aspects of roles, gaining information, and later insight into the roles as the tasks and behaviors become their own, but this is not a social imperative and is shaped by the individuals themselves [16].

The study highlights the importance of facilitators in developing comprehensibility, manageability (or bearability), and meaningfulness among family caregivers. All studied caregivers were women, which is practically the norm in most families in Kerala. Culturally, women's expected roles are marked with obligations to provide care, and prioritizing the provision of care over their own well-being is often normalized. This can often lead to helplessness, hopelessness, demoralization, and existential distress [18]. The identified enablers of comprehensibility provide the necessary expertise and assistance for family caregivers to plan patient-centered care goals while maintaining their own well-being. However, the lack of these enablers acts as a hindrance and could lead to caregiver distress and suffering. This lack of comprehensibility may lead to greater uncertainty, distress, and a sense of vulnerability among caregivers. The findings show that family caregivers manage their caregiving challenges with the necessary financial, informational, and emotional enablers. The lack of those might impair an individual's ability to properly manage their roles and responsibilities. We also found that caregivers find meaning and fulfillment in the caregiving role through its perceived benefits. Loss of these enablers can result in disputes in care relationships or caregiver issues that may even escalate to existential distress in extreme situations. This lack of meaning can cause significant suffering, resulting in the loss of their goals and objectives of life.

Understanding enabling and hindering aspects is crucial for targeted interventions and strategies for developing a high level of SOC in family caregivers. Developing interventions that promote these enablers can address family caregivers' challenges, reduce caregiving burden, and foster SOC. Given that Kerala's palliative care program is heavily dependent on family caregivers, there is a need for further research attention into how policymakers, healthcare providers, and family caregivers perceive these issues and explore how to use the concept of SOC to address barriers and leverage opportunities for preventing adverse caregiver outcomes.

Flexible job opportunities, accessible medical care, social support, financial assistance, and support in respite care can help develop a SOC among these Caregivers. Improving health literacy is critical to reducing the issues of family caregivers and obtaining help and resources. These suggestions seek to maximize enabling aspects while minimizing barriers for these family caregivers. Research indicates that factors such as effective communication, knowledge, social and financial support, and satisfaction in caregiving play crucial roles in alleviating the burden faced by family caregivers [19-21]. Additionally, cultivating a sense of coherence (SOC) has been identified as a key contributor to reducing this burden [6,12]. Therefore, possessing these elements enhances one's sense of coherence and diminishes the burden of caregiving. A good sense of coherence, in general, is correlated with and favors good pre-care relationships. Positive care patterns with successful adaptations are especially emphasized when prioritizing manageability and meaningfulness [22]. Caregivers with a low sense of coherence can be considered as possibly vulnerable to adverse outcomes for both the care recipient and the caregiver [23].

One limitation of the study was that we did not examine the theoretical salience of the concept of SOC before embarking on describing aspects related to it in our study setting. Also, we included only women caregivers aged between 38 and 58 years who were giving care for at least a year. We may have inadvertently selected caregivers who were "survivors" or could adapt to the circumstances. Including others who had just entered a caregiving role or male caregivers might have produced different results. Also, we did not include younger or older caregivers who may have experienced and responded to the same circumstances differently. We also did not consider potentially important aspects of the expression of SOC, such as the duration of caregiving, the attitude of the caregiver to the care recipient, and vice versa. These may limit the transferability of our findings to such situations.

Future research should explore these nuances along with cross-cultural variations. Kerala, characterized by a dearth of institutional care support, grapples with unique challenges, particularly with increasing spousal caregiving, making caregivers themselves older, given the prevalence of migration and nuclear family arrangements in the state. Understanding the specific dynamics in a broader, cross-cultural context will contribute to a more comprehensive and nuanced understanding of care dynamics, shedding light on how societal structures impact the experiences and challenges faced by caregivers. Other contexts in India and other low- and middle-income settings may have different demographic patterns, gender and power relationships, decision-making approaches, and meanings for suffering and care. There is also a need to look for cost-effective, scalable interventions in enhancing the SOC in family caregivers, which may have to be specific, such as therapies, psychoeducational interventions for caregivers, or broader salutogenic interventions, such as promoting positive mental health and well-being, such as those for healthy aging.

Moreover, investing in capacity building and skill-based training for healthcare providers is essential. This would enable them to better recognize distress among caregivers and provide timely and tailored support. While our findings offer limited insight in this direction, future research could further investigate the availability of health and social resources for caregivers, as well as their characteristics and unmet context-specific needs of these caregivers. Future studies can also examine the systemic inequalities in addressing caregiver needs, which is crucial for devising targeted programs at the health system level. This research can contribute to developing a comprehensive approach to supporting these caregivers and improving their overall well-being. Larger, diverse sample sizes and exploring other related concepts or variables such as caregiver burden, role conflicts, and caregiver burnout may provide a more comprehensive understanding of aspects influencing family caregivers, enabling more applicable findings. We did not explore the potential role of technology in developing SOC among caregivers, which may also be the focus of future research on this topic.

## Conclusions

Empowering the family caregivers of palliative care patients by enhancing the level of SOC can prevent or reduce their burden and improve the quality of care they provide. Various factors, including knowledge, access to resources, open communication, financial management plans, care approaches, and support networks, influence SOC's three components. Based on our findings, we feel that it is crucial to empower caregivers through comprehensive education and help them negotiate for more resources through the program or through peer groups and targeted assistance. We, therefore, advocate for improved resource accessibility, enhanced support networks, and policy and program review to improve support activities for family caregivers. By helping caregivers of palliative care enhance their sense of coherence levels, caregivers can be enabled to navigate their roles with resilience and find fulfillment in their caregiving journey.
